# Supplementary material for: Room-temperature pyro-catalytic hydrogen generation of 2D few-layer black phosphorene under cold-hot alternation
Source: Nat Commun. 2018 Jul 23;9:2889. doi: 10.1038/s41467-018-05343-w (PMC6056473; doi:10.1038/s41467-018-05343-w)
Supplement: Supplementary file 1 — Supplementary Information [file 41467_2018_5343_MOESM1_ESM.pdf]

H. You et al., "Room-temperature pyro-catalytic hydrogen generation of 2D few-layer black phosphorene under cold-hot alternation"

Supplementary Information

Supplementary Figures

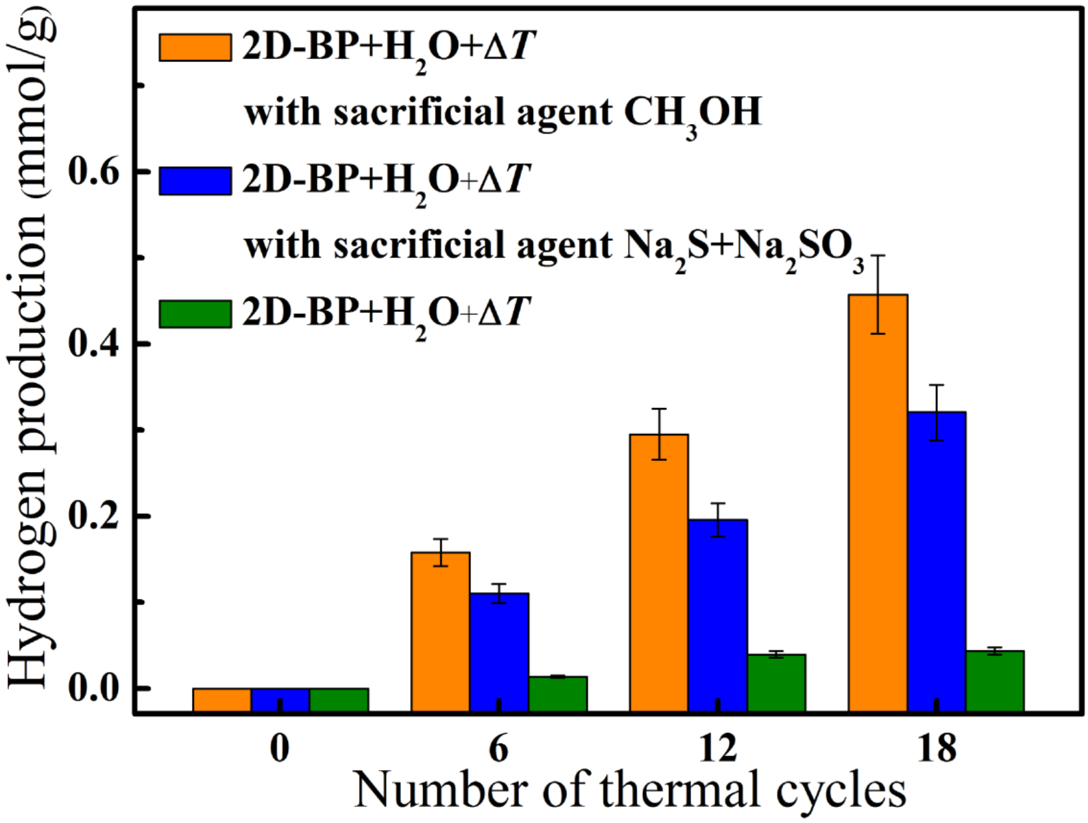

Supplementary Figure 1. The hydrogen evolution with/without the addition of different sacrificial agent.

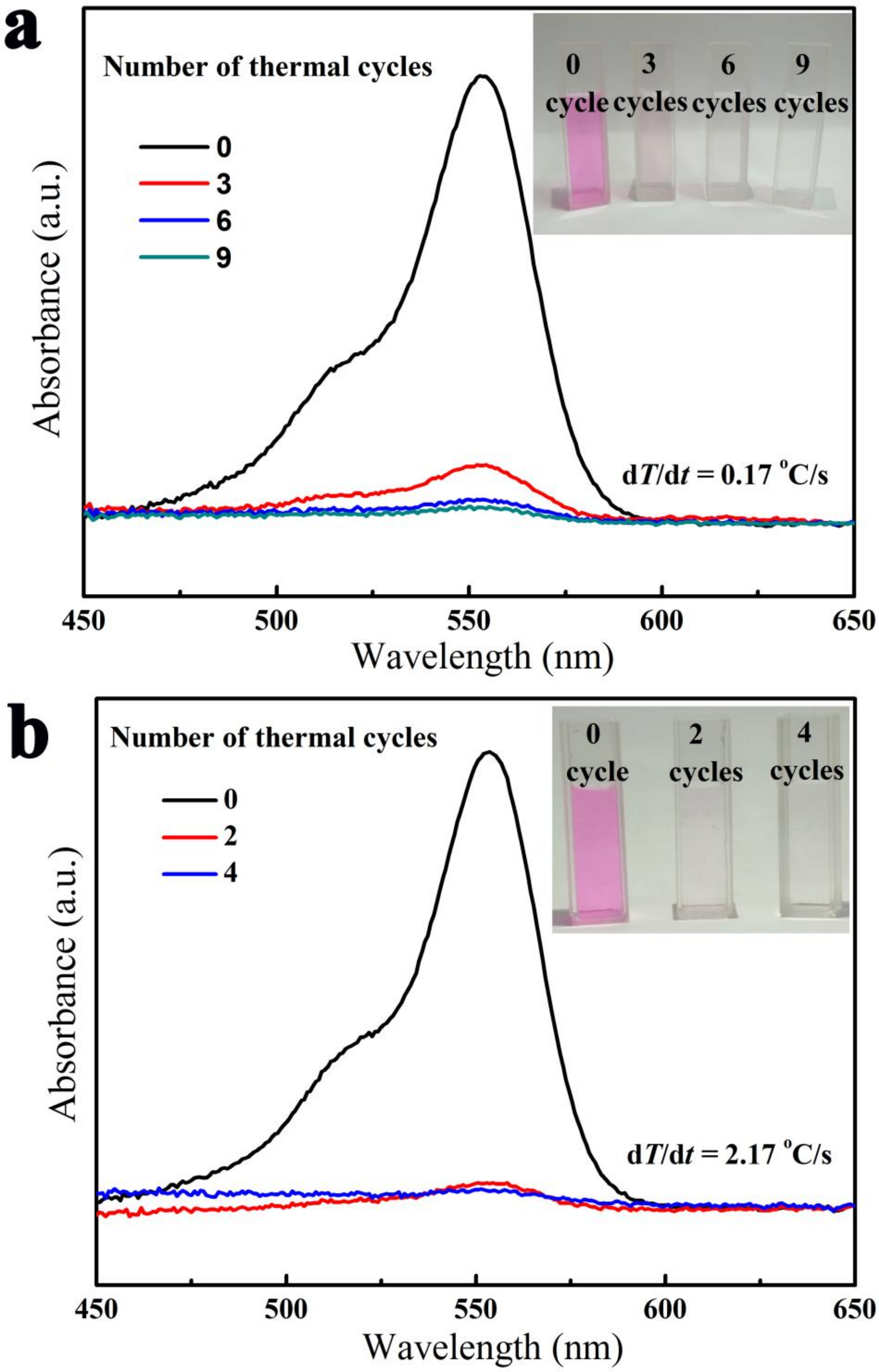

Supplementary Figure 2. The absorption spectra of RhB dye solution (5 mg/L) with the addition of 2D-BP after experiencing different thermal cycles with different d*T*/d*t*. a d*T*/d*t* of 0.17 ºC·s^{-1}; **b** d*T*/d*t* of 2.17 ºC·s^{-1}.

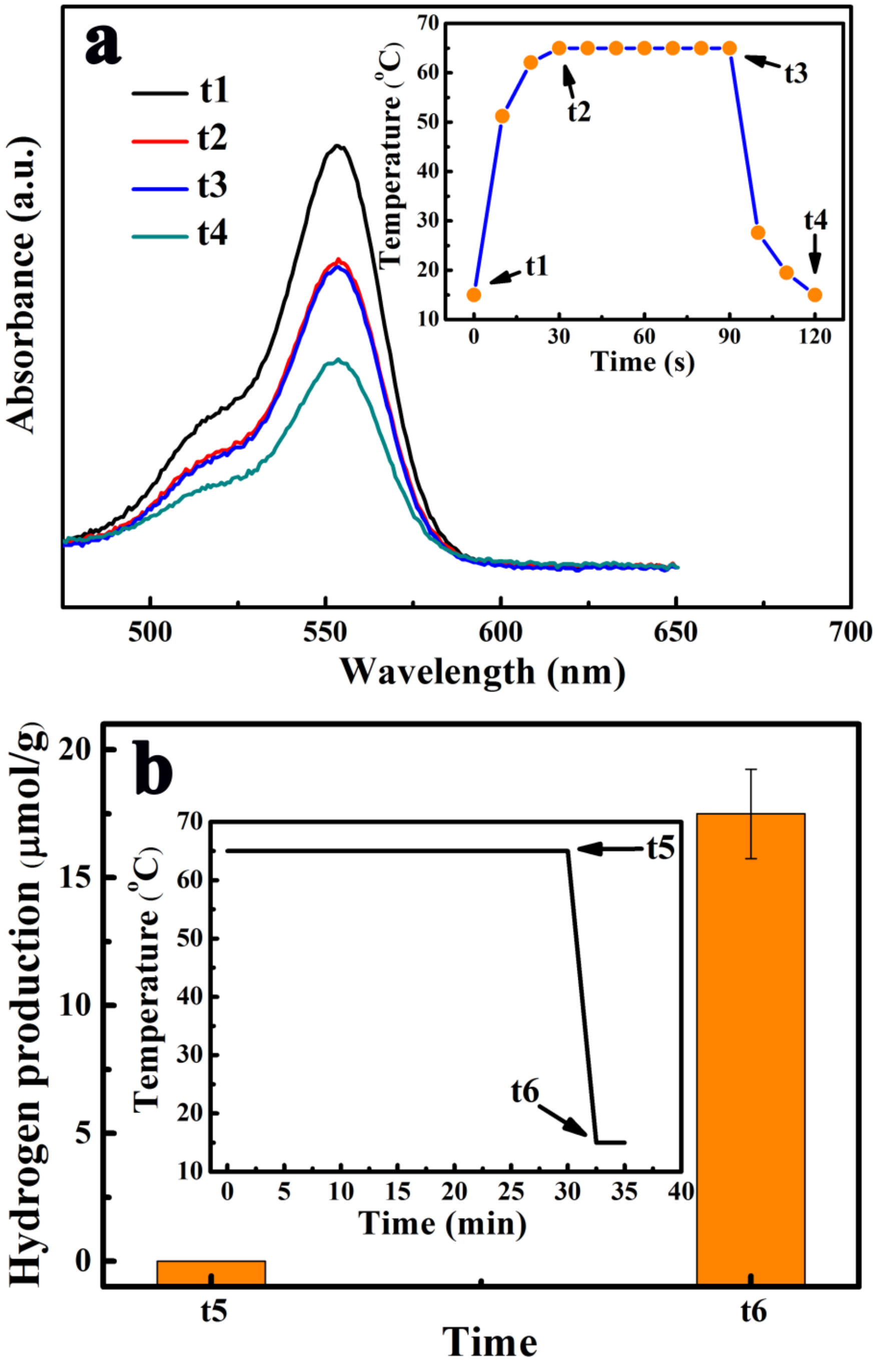

Supplementary Figure 3. Pyro-catalysis of 2D-BP in the decreasing temperature stage. a Dye decomposition. The inset is the decreasing temperature curve of dye decomposition. **b** hydrogen evolution experiment under temperature change. The inset is the temperature curve for the hydrogen production experiment. The *t*1, *t*2, *t*3, *t*4, *t*5 and *t*6 in (a) and (b) denote different temperature points.

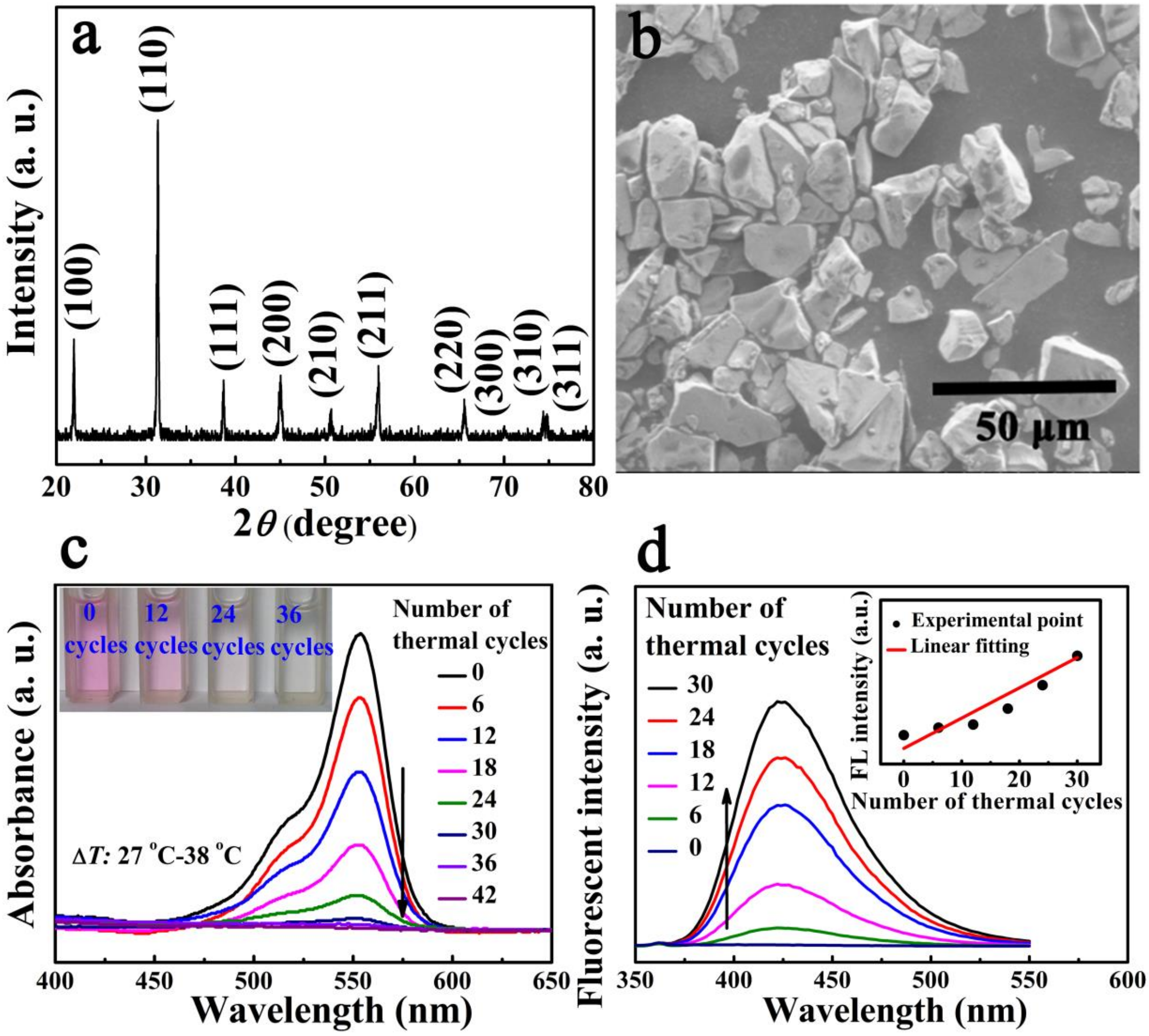

Supplementary Figure 4. $Pb(Mg_{1/3}Nb_{2/3})_{0.72}Ti_{0.28}O_3$ microcrystalline. **a** SEM. **b** XRD. **c** Pyro-catalytic absorption spectra of RhB dye solution. The inset is a dye decomposition photo. **d** Fluorescent (FL) spectra of 2-hydroxyterephthalic acid solution for trapping ·OH in the pyro-catalytic dye decomposition process. The inset shows the FL intensity at 425 nm against the 27-38 ^{o}C thermal cycles.

Supplementary Methods

In Supplementary Figure 2, the mixture of 1 mg 2D-BP and 50 mL RhB dye (5 $mg \cdot L^{-1}$) was put into a thin self-sealing bag and transferred between hot or cold bath every 30s.

In Supplementary Figure 4c, the reactive oxygen species of hydroxyl radical (·OH) in the pyro-catalytic dye decomposition can be detected by detecting the fluorescence signals of ·OH trapping agent terephthalic acid at 425 nm under the excitation at 315 nm. The strength of 2-hydroxyterephtalic acid PL peak is directly in proportion to the quantity of ·OH generated in the solution.
